# The New Ideals of District Nursing

**Published:** 1920-11-13

**Authors:** 


### The New Ideals of District Nursing.

The Central Council for District Nursing in London has already in its short term of existence taken a firm hold on the complex matters it was called into being to supervise. It has reduced overlapping, filled up untraversed areas, relieved congestion, and brought order out of something like chaos. It has now projected a new departure which is likely to have far-reaching consequences for good, and bids fair to revolutionise the nursing service in great towns.

In the memorandum which we reproduce in full on p. 154 the Council sets out that there are considerable numbers of patients requiring skilled nursing who are neither, strictly speaking, the patients of the District Nursing Associations established for the nursing of the sick poor in their own homes, nor are able to pay the full fees of a private nurse. Some live in flats or under circumstances which prevent them from providing board and lodging for a resident nurse. Many belong to the professional or commercial classes and cannot afford to pay the fees of a full-time nurse.

The case of these middle-class people in respect to both nursing and hospital treatment is being forced into prominence by the gradual shrinking of the "sick-poor" class and a continual increase in the number of those who cannot be so classified. The hospitals are now catering for their treatment in pay beds. We rejoice to learn that the Central Council proposes that their nursing be undertaken by visiting nurses under a central scheme in which the District Nursing Association shall be asked to participate.

The Council anticipates that the earnings of a visiting nurse should be approximately from four to six guineas a week, and the fees she would charge would vary from 2s. 6d. to 3s. 6d. a visit, with a reduction for double visits in one day or attendance by the week.

The whole matter resolves itself into one of organisation. Here are the patients, innumerable, glad to pay, often suffering much for want of attendance. Here are the nurses, looking out for just such work, which would secure them a certain amount of independence, a good income, and an interesting type of work, without the institutional atmosphere. The problem is to get them into communication without undue time being wasted in frequent journeyings from a distance. When that problem is solved, it will be a far less anxious business to tackle a case of serious illness than it is at present. The ideal is to have the nurse fully trained in all branches of nursing, available at a short distance from her *clientèle*, and competent to deal with every kind of call. So long as nurses cross and re-cross each other in their daily tracks there is a leakage in efficiency. The Central Council, which has already done so much to pull the various localities together in this respect, will not fail to go the best way to work.

